# Prevalence and Correlates of Health Risk Behaviors among University Students from a State in the Southern Region of Brazil

**DOI:** 10.3390/ijerph21050612

**Published:** 2024-05-11

**Authors:** Dartagnan Pinto Guedes, Keila Aparecida de Lima, Andre Luis dos Santos Silva

**Affiliations:** 1 Health Sciences Center, State University of Northern Parana, Jacarezinho 86400-000, Brazil; keiladelima9@gmail.com; 2 Health Department, Federal Technological University of Parana, Londrina 86036-370, Brazil; andre.luis2@hotmail.com

**Keywords:** lifestyles, surveys, university health, health promotion, public health, Brazil

## Abstract

Background: Surveys conducted in different regions of the world show that the prevalence rates of health risk behaviors (HRBs) in university students are sometimes higher than those found in non-university populations. This study aims to identify the prevalence rates and demographic and academic environment correlates associated with HRBs among Brazilian university students. Methods: In a cross-sectional epidemiological study, a random sample of 5310 university students answered an online questionnaire, with demographic (sex, age, skin color, marital status, and paid work) and academic setting information (housing type, size of campus, year, and shift of study), as well as items clustered in four HRB domains: personal safety and violence, sexual behavior and contraception, addictive substance use, eating habits, physical activity, and sleep. The data were analyzed statistically using bivariate analysis and hierarchical multiple regression. Results: The highest prevalence rates occurred in HRBs clustered in the domain of eating habits, physical activity, and sleep (>60%), while HRBs for personal security and violence were less prevalent (<15%). From 15% to 35% of university students assumed HRBs regarding addictive substance use, and approximately 50% reported risky sexual behavior. The university students most susceptible to HRBs were men, aged ≥ 22 years, living far from their family, studying on larger campuses, attending night classes, and with two or more years of study at the university. Conclusion: The findings suggest that policies and interventions in the university context aimed at students’ readiness to engage in a healthy lifestyle should target specific correlates associated with HRBs.

## 1. Introduction

Health behaviors refer to the set of individual behavior patterns that demonstrate some consistency across time, under more or less constant conditions, which, depending on their nature, may constitute protective or health risk components. In general, health behaviors result from the interaction of personal, environmental, and social dimensions, which emerge not only from the present but also from the individual’s past history. Moreover, these behaviors can vary according to cultural, ethnic, and religious attributes, and peer and family influence [1].

In this context, health risk behaviors (HRBs) refer to behavior patterns that increase the likelihood of health-related harm. An HRB may begin with the exploratory nature of the individual or the influence of the social environment, and it should be identified as early as possible, in order to minimize the consolidation of harmful practices, with important consequences for oneself and society [2]. In general, HRBs are clustered into four domains: (a) personal safety and violence, (b) sexual behavior, (c) addictive substance use, and (d) eating habits, physical activity, and sleep.

Worldwide, the vast majority of students begin their university studies before the age of 20, staying at the university for four or five years, constituting a crucial period for the acquisition and consolidation of health behaviors. The transition from high school to university education is a critical period, in which the achievement of independence by entering adulthood, the feeling of freedom in decision-making, the relative distance from family surveillance, and new friendships, relationships, and experiences contribute to greater vulnerability and exposure to various factors and situations that influence young people’s perceptions and attitudes [3]. However, this period also coincides with the moment when many risky behaviors are introduced or reinforced. These unhealthy behaviors have a negative impact not only during the time at university, but, in many cases, are maintained after this period, becoming highly harmful across the lifespan [4]. Therefore, there is growing recognition that university students are an important target-population for public health policymaking.

Surveys conducted in different regions of the world, including Europe [5,6], Asia [7,8], Africa [9,10], Latin America [11,12], and North America [13,14], showed that the prevalence rates of HRBs in university students are sometimes higher than those found in non-university populations. Unanimously, studies indicate that HRBs result from inadequate choices that, at different intensities and severity levels, negatively impact health, increasing the probability of an early diagnosis of chronic non-transmissible diseases and of mortality from external causes [15].

Particularly in Brazil, few studies have sought to investigate health behaviors in representative samples of the university population, demonstrating an important gap in the current knowledge. The studies identified to date have focused on isolated health behaviors, involving exclusive samples of specific courses, from a single institution, and with participants selected for convenience or another non-probabilistic method [12,16,17], thus, providing fragile inferences to support the selection and prioritization of HRBs and the implementation and continuous evaluation of interventions in this scenario.

Therefore, the objective of the current study was to identify the prevalence rates of HRBs self-reported by Brazilian university students and to establish associations with demographic and academic environment correlates. Publishing the findings is intended to encourage institutional policies to promote healthy lifestyles and structure more effective interventions aimed at groups more exposed to harmful health behaviors.

## 2. Materials and Methods

### 2.1. Study Design and Participants

An observational cross-sectional study was developed, following the STROBE (STrengthening the Reporting of OBservational studies in Epidemiology) guidelines [18]. Data analyzed were derived from the Health-Promoting University Project, a population-based cross-sectional study designed and implemented by the Federal Technological University of Paraná (UTFPR). To illustrate the size of the population pool addressed, the UTFPR attends approximately 30 thousand university students across 105 courses, distributed over 13 campuses, located in cities of different geographic regions in the state of Paraná, Brazil.

### 2.2. Sample

The sample size was established assuming an unknown success prevalence (*p* = 50%), 95% confidence level, and sampling error of three percentage points. However, considering that the sample planning involved clusters, a design effect equivalent to three was defined, and 20% was added to meet any loss in data collection indicating the minimum sample needed five thousand university students. However, the definitive sample used in the statistical processing consisted of 5310 university students. The sample was achieved by random draw with a three-stage cluster, namely, campus, course, and study year, with probability proportional to the size.

### 2.3. Study Variables

Data were collected through an online questionnaire known as the National College Health Assessment II (NCHA IIc), using an electronic platform via the web, accessed through desktops, notebooks, tablets, or smartphones, at any time and place of convenience and preference of the participants [19]. Currently, the NCHA IIc is widely used in international studies [20]. The printed format was translated and cross-culturally adapted following international recommendations [21] and its online format validated for use by Brazilian university students [22].

In addition to demographic data (sex, age, skin color, marital status, and paid work) and academic setting information (housing type, size of campuses, year and shift of study), we addressed items clustered into four HRB domains: (a) personal safety and violence; (b) sexual behavior and contraception; (c) addictive substance use; and (d) eating habits, physical activity, and sleep. The NCHA IIc question and answer options included in the study, along with the health risk behaviors definitions, are shown in Table 1.

### 2.4. Data Collection

The classroom chosen for this study was visited, and the research objectives and principles of secrecy, non-identification in the study, and non-influence on academic performance were explained to university students in order to complete the questionnaires. Subsequently, the university students were invited to participate in the study, and those who initially agreed received guidance and an individual password to access the electronic platform, thus confirming their anonymity. Participants were instructed to access the platform and self-complete the questionnaire within a deadline of seven days after release of the individual password. All participants’ rights were guaranteed by a Free and Informed Consent Term, signed electronically before the initiation of the NCHA IIc self-completion questionnaire in the online format.

The criteria adopted for the exclusion of any university student belonging to the selected classroom were as follows: (a) absence from classes on the day scheduled for the invitation to participate in the study and the distribution of the individual password to access the electronic platform; (b) refusal to participate in the study; (c) being subjected to any medical treatment or specific diet for weight loss or other health outcome; (d) pregnancy; (e) failure to complete the questionnaire on the electronic platform within seven days; and (f) age under 18 years or over 35 years.

### 2.5. Data Analysis

The data were processed with the computerized Statistical Package for the Social Sciences (SPSS^®^, version 26). Initially, descriptive statistics resources were used to characterize the sample by calculating the proportion of the distribution of university students in each stratum. The prevalence rates equivalent to the HRBs were shown in specific proportions (%), accompanied by the respective 95% confidence intervals (95% CI). To analyze the linearity of the associations between HRBs and potential correlates, the chi-square test (χ2) was used. Subsequently, the correlates that showed at least marginally significant associations (*p* ≤ 0.20) in the bivariate analysis were included in the multiple hierarchical regression modelling. In this case, the correlates were included in blocks; demographic data (block 1) were the first to be included in the model, followed by those related to the academic setting (block 2). All correlates with a statistical significance *p* < 0.05 remained in the multivariate model.

## 3. Results

Table 2 provides descriptive data on the sample selected in this study. Approximately one-third of the sample were women (38.2%), 39.1% were aged 21 to 25, 61.5% self-reported white, and 78.2% were single. In addition to studying, 23.1% of the university students analyzed reported part-time paid work, and 39.6% disclosed full-time paid work. Regarding data from the academic settings, 24.2% lived in student residences and 52.8% with their families; 21% of university students were from smaller campuses (<1500 students), and 37.7% from larger campuses (≥3000 students); 63.3% attended daytime classes, while the academic years were distributed similarly, that is, 32.2% in the first study year and 31.5% in the remaining years.

Table 3 shows the prevalence rates of the HRBs selected in this study according to the demographic and academic setting data. The most prevalent risk behaviors were in the domain of eating habits, physical activity, and sleep, followed by the sexual behavior and contraception domain. However, due to the extent of severity, the prevalence rates of risk behaviors observed in the domains of addictive substance use, personal safety, and violence are also worrying.

Regarding personal safety and violence, 9.4% (95% CI 8.7–10.1) of the university students reported not always wearing seat belts when driving a car or when traveling seated in the passenger seat; furthermore, among those who had ridden motorcycles, bicycles, or rollerblades in the previous 12 months, 8.6% (95% CI 8.0–9.2) did so without wearing a helmet. Approximately 12.4% (95% CI 11.4–13.4) reported having been involved in fights at least once in the previous 12 months, while 11% (95% CI 10.1–11.9) reported suicidal ideation at least once in their lives. In the domain of sexual behavior and contraception, 53.2% (95% CI 49.7–56.7) of the university students reported having had sexual intercourse at different times with ≥3 occasional partners in the previous year, and most of those who had performed sexual intercourse in the previous 30 days 50.9% (95% CI 47.6–54.2) did not use condoms or another protective barrier. In the case of addictive substance use, 35.4% (95% CI 32.5–38.3) admitted alcohol abuse, 25.6% (95% CI 23.7–27.5) used some type of illicit drug, and 13% (95% CI 11.9–14.1) were habitual tobacco users. Overall, 86.5% (95% CI 81.4–91.6) of the university students reported inadequate fruit/vegetable intake, 75.9% (95% CI 71.3–80.5) physical inactivity, and 56.4% (95% CI 52.7–60.1) insufficient sleep.

The univariate analysis showed that the overall prevalence rates of HRBs mask substantial variations between the strata of the correlates. From the list of potential correlates considered, sex and age were associated with all selected HRB items. Likewise, correlates corresponding to the university setting (housing type, size of campuses, academic year, and study shift) were found to be associated with most HRB items. However, marital status presented occasional associations, while skin color and paid work did not demonstrate significant correlates (*p* < 0.20).

The results of the hierarchical multiple regression are available in Table 4. Considering the demographic correlates, after adjustment by the other variables included in the model, we found significant associations between HRBs, sex, and age. As for the university setting, the four correlates considered remained significantly associated with specific HRBs.

Involvement in physical fights, smoking, alcohol abuse, risky sexual behavior, illicit drug use, and low fruit/vegetable intake were more frequent among men, while a higher prevalence of women reported suicidal ideation and physical inactivity. Younger university students were more frequently involved in physical fights, while their peers aged ≥ 21 years had a higher prevalence of risky sexual behavior, addictive substance use, low fruit/vegetable intake, and physical inactivity.

For the university setting correlates, the findings indicated that involvement in physical fights, risky sexual behavior, addictive substance use, and low fruit/vegetable intake were more prevalent among university students living in student residences. University students who studied on larger campuses, from the second year on, and who studied at night showed the highest prevalence of HRBs. The prevalence rates for involvement in physical fights, risky sexual behavior, addictive substance use, and low fruit/vegetable intake were significantly higher among university students who studied on larger campuses. Other than involvement in physical fights, suicidal ideation, and smoking, which were consistent throughout the time at university, the prevalence rates of the other HRBs increased as academic years progressed, while physical inactivity was more prevalent among university students studying at night.

## 4. Discussion

To the best of our knowledge, this is the first study to simultaneously report the prevalence rates of several HRBs and their associations with demographic and academic environment correlates in a representative sample of Brazilian university students. The study findings add new knowledge to the literature and provide important support for the design of interventions directed at health promotion in universities. The selected risk behaviors represent the most relevant contributors to the physical and mental health of the students in the present and across the lifespan [4].

Traditionally, a specific public health agenda has been built around HRBs, with particular interest in the transition phase between late adolescence and early adulthood [14]. In this context, the findings of this study provide solid evidence that the HRBs of university students are a cause for concern and, if maintained in post-university years, suggest that the population trends regarding the appearance and deterioration of chronic diseases associated with an unhealthy lifestyle are unlikely to change in future generations.

The current findings are important considering that the health behaviors of young adults are strong predictors of their health status at older ages [4]. In our sample, the most prevalent HRB was inadequate fruit/vegetable intake, followed by physical inactivity and insufficient sleep. Approximately ⅓ of university students reported alcohol abuse, and one in every group of four mentioned illicit drug use. The prevalence rates of risky behaviors in the personal safety/violence domain, especially involvement in physical fights and suicidal ideation, and in the sexual behavior and contraception domain, represented by the number of occasional partners and irregular use of condoms or another protective barrier, were also of concern. The significant link between HRBs with demographic and academic environment variables correlates shows that specific interventions are necessary to address the situation.

Regarding the personal safety and violence domain, both behaviors related to traffic safety were the least prevalent HRBs, showing broad approval for wearing seatbelts and helmets when riding a bicycle, motorcycle, or skating. In the current study, the prevalence rates of sporadic seatbelt and helmet use were around 9%, while in North American university students, these rates were close to 18% [13]. It should be noted, however, that the use of both pieces of personal safety equipment is mandatory under Brazilian law, which is not the case in the United States. Initially, sex and marital status were shown to be possible correlates of both HRBs (univariate analyses); however, when treated in the multivariate model through adjustment for confounders, the associations did not remain significant.

Specifically, concerning violence, 12.4% of the university students reported being involved in physical fights in the 12 months preceding the survey, with men, younger students, and those living in student residences engaging in this type of behavior more frequently. Physical aggression can be related to immaturity, the need for self-assertion and approval by peers, abusive alcohol and illicit drug use, and affective and loving factors [23]. As a benchmark for comparison, a survey involving university students from the United States and Mexico showed a prevalence of involvement in physical fights of 33.7%, 11.4% of which caused serious injuries, requiring medical services [24].

The reports of suicidal ideation, which are more frequent among women and reached 11% of the selected sample, are similar to those found in university students from other countries, both considering the female/male proportion and the overall prevalence [25]. According to psychiatric studies, most individuals who intend and/or attempt suicide find themselves in profound depression, with little hope in the world, the future, and themselves [26]. Considering the probable psycho-emotional suffering and physical health risk involved in these conditions, further studies should dedicate more attention to the determinants of these acts and propose preventive interventions, especially among women, who are the greatest victims of this problem.

Intense social relationships, dating, and affective discoveries mark the period at university. Therefore, this is a phase in which university students may intensify their sexual activity. Consequently, they are more exposed to contracting sexually transmitted diseases and may have to deal with an unwanted pregnancy. Among the consequences of an unwanted pregnancy are risks of complications in the case of attempted unsafe abortions, rejection of the baby after birth, economic implications, negative repercussions on their academic education, and even premature interruption of studies [27].

The sex risk behavior of university students selected in this study is worrying. Over half of the sample reported a higher number of occasional partners and irregular condom or other protective barrier use. Previous surveys conducted in Brazil identified similar proportions [12]; however, slightly lower prevalence rates were described in North American [13] and European [28] university students. As observed in other studies [28], male students who are older and reside in student residences admitted to sexual relationships with a higher risk. However, an original finding of this study was that first-year students reported safer sexual behavior when compared with their peers in subsequent years.

Despite the generally high schooling of the target population, misinformation, along with cultural components, may contribute to higher-risk sexual behavior. In this context, simply providing information does not seem to suffice as, in the field of health education, knowledge mastery may not always translate into safer practices [29]. Thus, even if it is a traditionally challenging intervention in the university environment, it is advisable that adequate information, through specific sex education programs that consider the pleasure and legitimacy of sex practice, be combined with techniques that help university students to improve their decision-making skills and incorporate a stance of self-responsibility.

Addictive substance use among university students that require abstention or restriction is a familiar topic in public health research. The period of studying at university is highly propitious to the appearance of disorders caused by smoking, alcohol, and illicit drug abuse, with serious consequences for future life [30]. In our sample, 13% of the university students were smokers, one of the lowest prevalence rates compared to other countries [5,7,8,10,13]. In a recent national survey, despite relevant regional differences, 22% of Brazilian university students reported regular tobacco use [16]. A review study with data from various countries showed smoking prevalence variations of 6% to 48% [11]. This wide variation may be due to how the habits of a society reflect specifically on the university population. Thus, if smoking is more common in a country, it is likely that the prevalence rates for smoking in the university population will also be higher. Therefore, the low prevalence rate observed in the present study may relate to the success of campaigns and actions against smoking in recent decades in Brazil, rendering the prevalence rate of smoking in the Brazilian population one of the lowest in the world [31]. Nevertheless, we recommend actions against smoking in the group of university students who adopt this HRB, considering that smoking is an important risk factor for some types of cancer and other chronic diseases [32].

In contrast, one out of three university students in our sample (35.4%) reported the abusive consumption of alcoholic beverages. This proportion is higher than that found in a national survey carried out in Brazil (18%) [16], but coincides with results from some Latin American [11] and European [33] countries and is lower than the prevalence rates found in North American [13] and Australian [34] universities. These data confirm that alcohol is the most commonly consumed addictive substance by university students around the world and may be associated with other HRBs, such as involvement in physical fights, unprotected sexual intercourse, smoking, illicit drug use, physical inactivity, and an unbalanced diet [35]. Alcoholic is used as a strategy for relaxation, stress relief, and the strengthening of social bonds among university students, and colleagues and the media encourage its consumption at university gatherings. However, chronic abuse may jeopardize life projects, family and social coexistence, and physical and mental health [36].

Illicit drug use is perceived as an attitude that challenges social norms and rules. The abusive consumption of alcoholic beverages, like illicit drugs, can expose users to other HRBs, even if the use is sporadic and associated with supposedly favorable contexts, such as social gatherings. The alarming prevalence of illicit drug use over the previous 30 days (25.6%) was similar to that found in Brazilian university students in a national survey (25.9%) [16]. These data corroborate surveys with university populations from European [6], African [10], and Latin American [11] countries, as well as the United States [13]. This finding points to the need for prevention strategies and adequate public policies, considering the strategic function of universities as a center that produces knowledge and trains leaders who influence social and health behaviors.

Addictive substance use was strongly associated with multiple demographic and university environment correlates. These findings allow for the identification of the strata of the university population most likely to consume these substances, helping to target appropriate interventions to the most vulnerable segments. Corroborating the results of previous studies [6], male and older university students presented an increased likelihood of consuming addictive substances. As the literature shows, males are probably more likely to identify with addictive substance use due to social influence and cultural factors demonstrating power. Similarly, with advancing age, university students may have more years of independent life, and, in the absence of more health-promoting guidance, become more vulnerable to addictive substance use [37].

Housing type, campus size, and academic year also represented important mediators for addictive substance use. In this case, it is possible that university students living with their families have more difficulty in overcoming protection barriers naturally imposed by the support and control of family members, which is not the case when they live in student residences, thus exposing them more intensely to smoking, alcohol, and illicit drug use [6]. Specifically, in the larger campuses and from the second study year on, higher prevalence rates of alcohol abuse and illicit drug use were identified. Speculatively, considering that the larger campuses are located in cities with larger populations, the disparity found among campuses with >3000 university students compared to smaller campuses may be indicative that urbanization impacts both HRBs. On the other hand, higher levels of alcohol abuse and illicit drug use as the years of study progress may result from the progressive adaptation and integration of students in the university environment, associated with peer influence and the need to be part of a group. These findings show that specific intervention actions are necessary and focused on these three correlates of the university environment.

Another finding from this study was the high prevalence of sleep problems (56.4%), which coincides with results from other studies available in the literature [38]. Sleep-related issues have been intensively investigated in recent decades, and have been shown to be associated with multiple morbidities, such as obesity, hypertension, diabetes, mental disorders [39], and other HRBs, including smoking, alcohol abuse, inadequate fruit/vegetable intake, and physical inactivity [40]. Furthermore, sleep is considered particularly important for learning and memory and has implications for emotional regulation and behavior. More specifically, poor-quality and insufficient sleep duration are related to deficiencies in the processing of received stimuli and in the ability to concentrate, which, in turn, translates into compromised academic performance [41]. It is interesting to note that, unlike the findings of some studies in other countries [42], but coinciding with observations in a study involving Brazilian university students [43], no significant associations were observed between insufficient sleep and the demographic and university environment correlates considered in the present study. Initially, through bivariate analysis, sex, age, housing type, and study shift seemed probable correlates of insufficient sleep; however, the multivariate analysis, after adjustments by the other correlates, rendered this untrue.

Similar to what was observed in a prior survey conducted in Brazil [12] and other countries [5,6,7,8,9,10,11,12,13,14], the results showed that insufficient fruit/vegetable intake was the most prevalent HRB among the university students selected for this study (86.5%). An eating pattern composed of low fruit/vegetable consumption is associated with an increased risk of cardiometabolic diseases and some types of cancer [44], thus indicating that the eating habits of university students deserve special attention. The entrance to university, along with poor food education in adolescence, and the lack of time to enjoy complete meals due to increased activities and academic occupations, lead students to inadequate food choices, primarily characterized by replacing complete meals with practical and fast snacks of low nutritional value [45]. On this theme, an experimental intervention focused on offering university students nutritional knowledge related to disease prevention and healthy food alternatives to increase fruit/vegetable intake showed positive results [46].

In line with the results of previous studies, inadequate fruit/vegetable intake was more prevalent among males, which may indicate a trend towards healthier eating habits in females [6,12]. However, different from the findings of some studies [9,10], older university students had a higher prevalence of inadequate fruit/vegetable intake. In fact, a greater interest in food-related issues may lead to greater concern in consuming healthier foods, and being culturally responsible for the preparation of meals may favorably influence the food choices of females [47], thus justifying the sex differences observed in the consumption of the recommended portions of fruits/vegetables.

Regarding age, it is possible that lower fruit/vegetable intake at later ages may be a consequence of higher exposure to the prevailing food pattern in today’s society, which includes higher amounts of processed and animal-based foods. Corroborating previous findings [48,49], university students who lived with their families reported higher fruit/vegetable intake when compared to their peers who lived far from their family. In this sense, previous studies on the impact of the family style in terms of eating habits revealed that few university students maintained the same eating habits they were used to before going to university, while the majority reported that, faced with the new routine and study environment, they started to adopt worse eating practices [50]. In addition, campus size and study year were identified as correlates of the university environment associated with fruit/vegetable intake, which suggests the need to focus attention on university students from larger campuses and in the final years of study as a target group in interventionist actions aimed at healthy eating practices.

Another important HRB is physical inactivity. The results showed that most university students included in this study were considered inactive (75.9%). In a study with university students from 23 countries, with different economic and sociocultural contexts, the prevalence of physical inactivity varied from 33% in European countries to 44% in less developed countries [51]. A meta-analysis identified that the global prevalence of physical inactivity in North American university students is close to 50% [52]. A systematic review carried out in Brazil indicated physical inactivity in about 40% of university students [17]. In comparison with available data from the Brazilian population in general, a recent survey showed that 49.4% of young adults between 18 and 24 years old were insufficiently active [53]. Thus, despite being referred to as a relevant risk behavior for health hazards [54], the prevalence of physical inactivity found in this study was higher than the rates available in the literature, confirming the need for actions that encourage the adoption of a more active and healthy lifestyle. Moreover, adequate and sufficient physical activity presents important clusters with the adoption of other healthy behaviors, such as areduction in smoking, adequate eating habits, and stress control [55].

Studies have pointed out that the shortage of adequate spaces and difficulty in organizing time devoted to academic activities, exercise, and sport are the main barriers to the practice of physical activity, due to the long time spent in institutions, as for some, the university setting becomes an extension of their residence [56]. The positive perception of the university setting, social support of friends, and preference for active leisure are also fundamental psycho-emotional attributes for the adherence to physical activity by university students [57].

When analyzing the demographic correlates of physical inactivity, consistent with what is available in the literature [17,51,52], sex and age showed significant associations with physical inactivity. Women were usually more inactive than men, and older university students were progressively more likely to be physically inactive than their younger peers. This finding might be related to the fact that older university students, as a rule, are more intensely involved with academic obligations and occupational activities, so they present less interest and expectation in entertainment that requires some sort of physical effort and interact with their peers through more sedentary social behavior [52]. This is worrisome because evidence shows that the level of physical activity in the final year of university is predictive of the physical activity habit in the future after completion of the university period [58].

The reasons for the differences in the prevalence rates of physical inactivity between the sexes are unclear. However, previous studies have referred to combinations of sociocultural and biological factors that may or may not engage women and men in physical activity. The higher involvement of men can be explained, in part, by the fact that, from an early age, they have been encouraged to partake in activities with high physical demands, while women are directed to expressive and physically more passive activities. Similarly, more effective participation of men in physical activity may result from a greater amount of positive reinforcement and encouragement for their practice, received since childhood [59].

Data stratified by correlates of the university environment showed that the prevalence of physical inactivity remained stable on campuses of different sizes, suggesting that this HRB is not restricted solely to large urban centers. However, university students in the more advanced years of study and the night shift had greater chances of association with exposure to physical inactivity. Some speculation on these results may be that most university students with the night shift are involved with work occupations during the day and, thus, have less time available to perform physical activity through exercise, sport, and leisure. In the present study, 74.6% of the university students on the night shift reported paid work during the day, while only 22.3% on the day shift reported this activity.

Among the limitations of this study, it is noteworthy that the investigation method employed involves self-reported responses, thus allowing for possible memory bias or even biased statements towards what is considered desirable. However, self-reporting is the current procedure in studies such as this one, as it is the most viable method of gathering data in population-based surveys. Certain common procedures that minimize this limitation were adopted here: anonymous questionnaire, voluntary participation, filling out the questionnaire without the presence of the researchers, and the guarantee of the confidential nature of the information provided. In addition, the large sample size, to some extent, minimizes any inaccuracy in the calculated estimates. Moreover, the cross-sectional approach of the data does not allow for inferences of causality in the association between HRBs and the investigated correlates, increasing the risk of a reverse causality bias. Therefore, the identified associations should not be considered conclusive, and further longitudinal studies are needed to address this limitation, which are currently being conducted by the authors of this manuscript.

The main strengths of this study relate to the concept, design, and conduct of the Health-Promoting University Project. The project meets a comprehensive cultural and geographic diversity and provides robust and up-to-date data on the HRBs of university students from a representative state in Southern Brazil, which enables generalization of the results to a larger population universe. The findings may add new evidence to the scarce body of knowledge about the prevalence of HRBs and the associated demographic and university environment correlates, considering that studies involving Brazilian university students and those from other regions in the world are rare. Since this population is composed of young adults, it is important to identify these behaviors at an early stage and invest heavily in prevention and control. Regarding methodology, possible seasonal interferences in the reports of university students were minimized as data collection was carried out over a short period (three months) and in the same season of the year (spring), which, along with a minimum refusal rate to participate in the study, ensures greater reliability of the findings.

## 5. Conclusions

In conclusion, the highest prevalence rates were identified in the HRBs grouped in the domain of eating habits, physical activity, and sleep (>60%). The HRBs in the domain of personal safety and violence were less prevalent (<15%). Between 15% and 35% of university students assumed HRBs for addictive substance use, and about 50% reported risky sexual behavior. The findings allowed us to trace the groups of university students most susceptible to HRBs. The most noteworthy are males, age ≥ 22, living far from their family, studying on campuses of ≥3000 students, studying at night, and having studied at the university for two or more years.

The implications of these findings are relevant for the public health area, as the high prevalence of HRBs represents a key element for future investigations to intervene in and reverse this trend and minimize the present and future risk of the subsequent appearance of diseases derived from harmful behavior. Modern universities are home to a large portion of the young adult population, and monitoring systems and interventions aimed at health promotion should be an institutional commitment and part of the organization and services provided to the student community. The period at university may be an ideal time to address health education and the readiness of students to engage in health protection practices. The fact that many university students later become multipliers and role models in society makes this task even more important.

## Figures and Tables

**Table 1:** Questions, answer options, and definitions of health risk behaviors used in these study.

Questions	Answer Options	Risk Definitions
Personal security and violence		
(a) Within the last 12 months, how often did you wear a seatbelt when you rode in a car?	“Never”, “rarely”, “sometimes”, “most of the time”, “always”.	“Never”, “rarely”, “sometimes”.
(b) Within the last 12 months, how often did you wear a helmet when you rode a bicycle/motorcycle/skating?	“Never”, “rarely”, “sometimes”, “most of the time”, “always”.	“Never”, “rarely”, “sometimes”.
(c) Within the last 12 months were you in a physical fight?	“No”, “yes”.	“Yes”
(d) Have you ever seriously considered suicide?	“No”, “yes”.	“Yes”
Sexual behavior and contraception		
(a) Within the last 12 months, with how many partners have you had oral sex, vaginal intercourse, or anal intercourse?	Participants pointed out the number of partners.	≥3 partners.
(b) Within the last 30 days, how often did you or your partner(s) use a condom or other protective barrier (e.g., male condom, female condom, dam, glove) during oral sex, vaginal intercourse, or anal intercourse?	“Never”, “rarely”, “sometimes”, “most of the time”, “always”.	“Never”, “rarely”, “sometimes”.
Addictive substance use		
(a) Within the last 30 days, on how many days did you use tobacco and derivatives (e.g., cigarettes, e-cigarettes, hookah, cigars, smokeless tobacco)?	“No days”, “1–2 days”, “3–5 days”, “6–9 days”, “10–19 days”, “20–29 days” “daily”.	Any frequency of use.
(b) Over the last two weeks, how many times have you had ≥5 drinks of alcohol at a sitting (e.g., beer, wine, distilled drinks)?	Participants pointed out the number of times.	Any frequency of use.
(c) Within the last 30 days, on how many days did use illicit drugs (e.g., marijuana, cocaine, opiates, inhalants, ecstasy)?	“No days”, “1–2 days”, “3–5 days”, “6–9 days”, “10–19 days”, “20–29 days”, “daily”.	Any frequency of use.
Eating habits, physical activity, and sleep		
(a) How many servings of fruits and vegetables do you usually have per day (1 serving = 1 medium piece of fruit; ½ cup fresh, frozen, or canned fruits/vegetables; ¾ cup fruit/vegetable juice; 1 cup salad greens; or ¼ cup dried fruit?	“No servings per day”, “1–2 servings per day”, “3–4 servings per day”, “≥5 servings per day”.	<5 servings per day.
(b) On how many of the past 7 days did you do physical activity of moderate to vigorous intensity (caused a noticeable or large increase in breathing or heart rate, such as a brisk walk or jogging) for at least 30 min?	Participants pointed out the number of days, from none to 7 days.	<5 days per week.
(c) On how many of the past 7 nights did you get enough sleep so that you felt rested when you woke up in the morning?	Participants pointed out the number of nights, from none to 7 nights.	<5 nights per week.

**Table 2:** Descriptive data for the sample selected in the study (n = 5310).

Indicators	n (%)	Indicators	n (%)
Demographic data		University setting data	
Sex		Housing type	
Female	2029 (38.2%)	Student residence	1285 (24.2%)
Male	3281 (61.8%)	Parent’s home	2804 (52.8%)
Age		Homestay	1094 (20.6%)
≤20 years	1543 (29.1%)	Single home	127 (2.4%)
21–25 years	2078 (39.1%)	Campus size	
≥26 years	1689 (31.8%)	<1500 students	1113 (21,0%)
Skin color		1500–3000 students	2195 (41.3%)
White	3266 (61.5%)	>3000 students	2002 (37.7%)
black	2044 (38.5%)	Academic year	
Marital status		1st	1711 (32.2%)
Single	4152 (78.2%)	2–3rd	1929 (36,3%)
Married	1158 (21.8%)	4th or more	1670 (31.5%)
Paid work		Study shift	
No work	1980 (37.3%)	Day	3362 (63.3%)
Part-time	1227 (23.1%)	Night	1948 (36.7%)
Full-time	2103 (39.6%)		

**Table 3:** Prevalence rates (95% CI) of health risk behaviors according to each demographic correlate and academic setting of Brazilian university students.

	Personal Security and Violence				Sex Behavior and Contraception	
	Don’t always Wear a Seatbelt	Don’t always Wear a Helmet	Involvement in Physical Fight	Suicidal Ideation	≥3 Occasional Partners Per Year	Don’t always Use Protective Barrier
Overall	9.4 (8.7–10.1)	8.6 (8.0–9.2)	12.4 (11.4–13.4)	11.0 (10.1–11.9)	53.2 (49.7–56.7)	50.9 (47.6–54.2)
Demographic data						
Sex	*p* = 0.152	*p* = 0.168	*p* < 0.001	*p* = 0.001	*p* = 0.001	*p* < 0.001
Female	6.5 (6.0–7.0)	5.8 (5.3–6.3)	6.7 (6.1–7.3)	14.9 (13.8–16.0)	43.1 (40.0–46.2)	42.8 (39.7–45.9)
Male	11.2 (10.4–12.1)	10.3 (9.5–10.1)	15.9 (14.7–17.1)	8.6 (7.9–9.3)	55.7 (52.1–59.3)	59.6 (55.4–63.7)
Age	*p* = 0.183	*p* = 0.191	*p* = 0.001	*p* = 0.025	*p* = 0.003	*p* < 0.001
≤20 years	11.5 (10.7–12.4)	10.1 (9.3–10.9)	17.1 (16.0–18.2)	8.8 (8.1–9.5)	58.8 (55.0–62.6)	44.1 (41.1–47.1)
21–25 years	9.9 (9.2–10.7)	9.1 (8.4–9.9)	12.6 (11.7–13.5)	10.2 (9.4–11.1)	50.3 (47.1–53.5)	54.3 (50.7–58.0)
≥26 years	7.4 (6.8–8.1)	6.9 (6.3–7.5)	8.9 (8.2–9.6)	13.9 (12.9–15.0)	46.6 (43.4–49.8)	57.5 (53.7–61.3)
Skin color	*p* = 0.461	*p* = 0.574	*p* = 0.425	*p* = 0.057	*p* = 0.238	*p* = 0.242
White	8.9 (8.2–9.6)	8.3 (7.6–9.0)	11.8 (10.9–12.9)	11.6 (10.7–12.5)	49.2 (46.0–49.4)	51.5 (48.2–54.8)
black	10.1 (9.3–10.9)	9.1 (8.3–9.9)	13.4 (12.4–14.4)	10.2 (9.4–11.0)	53.6 (50.0–57.2)	55.8 (52.2–59.4)
Marital status	*p* = 0.316	*p* = 0.328	*p* = 0.276	*p* = 0.059	*p* = 0.067	*p* = 0.084
Single	10.1 (9.4–10.8)	9.3 (8.6–10.0)	13.2 (12.2–14.2)	11.3 (10.4–12.2)	49.3 (46.2–52.4)	54.7 (51.1–58.3)
Married	7.0 (6.3–7.7)	6.1 (5.6–6.7)	9.6 (8.8–10.5)	9.9 (9.1–10.7)	56.8 (60.5–60.5)	47.9 (44.7–51.2)
Paid work	*p* = 0.442	*p* = 0.438	*p* = 0.210	*p* = 0.146	*p* = 0.261	*p* = 0.258
No work	10.3 (9.5–11.1)	9.5 (8.8–10.2)	14.8 (13.7–15.9)	10.3 (9.4–11.2)	49.6 (46.4–52.8)	51.1 (47.8–47.8)
Part-time	9.1 (8.3–10.0)	8.4 (7.7–9.1)	11.9 (11.0–12.9)	10.8 (9.9–11.7)	49.2 (46.1–52.3)	53.1 (49.6–56.6)
Full-time	8.8 (8.1–9.6)	7.9 (7.2–8.7)	10.5 (9.6–11.5)	11.8 (10.8–12.8)	53.1 (49.6–56.6)	55.2 (51.5–59.0)
University setting data						
Housing type	*p* = 0.583	*p* = 0.592	*p* < 0.001	*p* = 0.038	*p* = 0.001	*p* = 0.002
Student’s residence	9.2 (8.4–10.1)	8.4 (7.7–9.1)	18.0 (16.7–19.3)	12.6 (11.6–13.6)	59.3 (55.2–63.4)	60.4 (56.2–64.6)
Parent’s home	9.5 (8.7–10.3)	8.6 (7.9–9.3)	9.1 (8.3–9.9)	9.8 (9.0–10.6)	46.5 (43.3–50.1)	48.8 (45.6–52.1)
Homestay	9.3 (8.5–10.2)	8.9 (8.2–9.6)	14.4 (13.3–15.6)	11.9 (10.9–12.9)	52.2 (48.8–55.6)	57.1 (53.4–60.8)
Single home	9.9 (9.1–10.7)	8.3 (7.6–9.1)	12.9 (11.9–14.0)	13.8 (12.7–15.0)	50.7 (47.5–53.9)	47.7 (44.5–50.9)
Campus size	*p* = 0.568	*p* = 0.441	*p* = 0.414	*p* = 0.087	*p* = 0.214	*p* = 0.207
<1500 students	10.0 (9.2–10.8)	9.6 (8.8–10.4)	13.9 (12.8–15.0)	10.0 (9.2–10.8)	48.1 (44.9–51.3)	50.8 (47.5–54.1)
1500–3000 students	9.2 (8.3–10.2)	8.4 (7.6–9.2)	12.4 (11.4–13.4)	10.4 (9.5–11.3)	50.6 (47.4–53.8)	52.5 (49.1–49.1)
>3000 students	9.3 (8.5–10.1)	8.3 (7.5–9.1)	11.6 (10.7–12.5)	11.9 (10.8–13.1)	52.8 (56.2–56.2)	55.3 (51.6–59.0)
Academic year	*p* = 0.614	*p* = 0.562	*p* = 0.418	*p* = 0.024	*p* =0.013	*p* < 0.001
1st	9.8 (9.0–10.6)	9.1 (8.3–9.9)	13.1 (12.2–14.1)	9.8 (9.0–10.7)	57.3 (53.6–61.0)	43.9 (40.8–39.7)
2–3rd	9.4 (8.6–10.2)	8.5 (7.7–9.3)	12.6 (11.6–13.6)	10.7 (9.8–11.6)	51.0 (47.7–54.3)	54.4 (50.8–58.0)
4th or more	9.2 (9.4–10.1)	8.4 (7.6–9.2)	11.6 (10.6–12.7)	12.4 (11.3–13.5)	46.1 (42.9–42.9)	58.1 (54.3–54.3)
Study shift	*p* = 0.726	*p* = 0.618	*p* = 0.362	*p* = 0.053	*p* = 0.285	*p* = 0.226
Day	9.3 (8.6–10.0)	8.4 (7.6–9.2)	11.5 (10.6–12.4)	10.4 (9.5–11.3)	49.6 (46.4–52.8)	51.7 (48.3–55.1)
Night	9.6 (8.8–10.5)	9.0 (7.2–9.9)	13.9 (12.9–14.9)	12.1 (11.1–13.1)	53.1 (49.6–56.6)	55.8 (52.1–59.5)
	Addictive Substance Use			Eating Habits, Physical Activity, and Sleep		
	Smoking	Binge Drinking	Illicit Drugs	<5 Servings of Fruits and Vegetables	Physical Inactivity	<5 Nights per Week Slept Enough
Overall	13.0 (11.9–14.1)	35.4 (32.5–38.3)	25.6 (23.7–27.5)	86.5 (81.4–91.6)	75.9 (71.3–80.5)	56.4 (52.7–60.1)
Demographic data						
Sex	*p* = 0.003	*p* < 0.001	*p* < 001	*p* < 0.001	*p* = 0.012	*p* = 0.108
Female	9.4 (8.5–10.5)	20.4 (18.8–22.0)	16.5 (15.3–17.7)	78.3 (73.5–83.1)	82.6 (77.3–88.0)	52.6 (49.2–56.0)
Male	15.2 (13.9–16.5)	44.7 (41.4–48.0)	31.3 (28.8–33.8)	91.6 (86.3–96.9)	71.7 (67.4–76.2)	58.7 (54.9–62.5)
Age	*p* = 0.019	*p* = 0.012	*p* = 0.007	*p* = 0.013	*p* = 0.019	*p* = 0.162
≤20 years	8.9 (8.1–9.8)	28.8 (26.5–3.1)	17.1 (15.8–18.4)	78.1 (73.3–82.9)	70.8 (66.6–75.1)	53.9 (50.3–57.5)
21–25 years	13.8 (12.6–15.1)	36.1 (33.2–39.0)	27.6 (25.5–29.7)	89.3 (84.1–94.5)	75.2 (70.6–79.9)	55.7 (52.0–59.4)
≥26 years	14.5 (13.2–15.8)	38.6 (35.5–41.8)	28.8 (26.5–26.5)	87.6 (82.5–92.7)	80.7 (75.6–86.0)	59.4 (55.3–63.5)
Skin color	*p* = 0.626	*p* = 0.404	*p* = 0.368	*p* = 0.397	*p* = 0.328	*p* = 0.276
White	13.3 (12.2–14.4)	34.5 (31.6–37.4)	24.4 (22.6–26.2)	87.1 (82.2–92.2)	74.7 (70.1–79.3)	54.9 (51.2–58.6)
black	12.8 (11.7–13.9)	36.9 (33.9–40.0)	27.6 (25.4–25.4)	85.6 (80.5–90.7)	77.1 (72.3–82.0)	58.7 (54.8–62.6)
Marital status	*p* = 0.083	*p* = 0.036	*p* = 0.074	*p* = 0.058	*p* = 0.158	*p* = 0.304
Single	12.0 (10.9–13.1)	37.5 (34.5–40.6)	27.2 (25.1–29.3)	88.7 (83.4–94.0)	74.6 (70.1–79.2)	55.7 (52.0–59.4)
Married	14.8 (13.3–16.3)	27.8 (25.6–30.1)	19.9 (18.4–21.4)	78.8 (74.0–83.7)	80.4 (75.3–85.7)	58.9 (54.8–63.0)
Paid work	*p* = 0.418	*p* = 0.341	*p* = 0.438	*p* = 0.185	*p* = 0.237	*p* = 0.213
No work	12.3 (11.2–13.4)	33.8 (31.0–36.6)	24.8 (22.9–26.8)	84.0 (79.0–89.1)	74.8 (70.2–79.5)	54.1 (50.5–57.7)
Part-time	12.8 (11.7–13.9)	35.6 (32.7–38.5)	26.9 (24.1–29.1)	85.8 (80.7–90.9)	75.7 (71.1–80.4)	55.7 (52.0–59.4)
Full-time	13.8 (12.6–15.0)	36.8 (33.8–39.9)	25.6 (23.6–27.7)	89.2 (84.0–94.4)	76.9 (72.2–81.7)	58.9 (54.9–63.0)
University setting data						
Housing type	*p* = 0.002	*p* < 0.001	*p* < 0.001	*p* < 0.001	*p* = 0.125	*p* = 0.171
Student’s residence	16.9 (15.4–18.4)	42.1 (39.0–45.2)	34.6 (31.7–37.5)	94.4 (89.4–99.5)	79.0 (74.0–84.1)	59.4 (55.3–63.5)
Parent’s home	10.2 (9.3–11.1)	31.2 (28.7–33.7)	20.8 (19.1–22.5)	79.6 (74.7–84.5)	73.2 (68.8–77.8)	54.3 (50.7–57.9)
Homestay	15.8 (14.4–17.2)	38.4 (35.4–41.5)	27.4 (25.3–29.6)	93.7 (88.6– 8.8)	79.2 (74.3–84.2)	57.9 (54.1–61.7)
Single home	12.2 (11.1–13.4)	36.1 (33.2–39.0)	26.7 (24.6–28.9)	89.8 (84.7–94.9)	76.9 (72.3–81.7)	60.1 (55.9–64.3)
Campus size	*p* = 0.104	*p* = 0.021	*p* = < 0.001	*p* = 0.031	*p* = 0.364	*p* = 0.209
<1500 students	10.6 (8.9–11.5)	29.7 (27.3– 32.0)	19.8 (18.3–21.3)	79.3 (74.5–84.0)	74.8 (70.2–79.5)	53.8 (50.3–57.3)
1500–3000 students	12.2 (11.1–13.3)	34.5 (31.6–37.4)	23.1 (21.3–24.9)	86.2 (81.1–91.3)	76.2 (71.5–81.0)	56.2 (52.5–59.9)
>3000 students	15.2 (13.9–16.5)	39.5 (36.4–42.6)	31.6 (29.0–34.2)	90.9 (85.7–96.1)	76.6 (71.9–81.3)	58.0 (54.2–61.8)
Academic year	*p* = 0.044	*p* = 0.029	*p* < 0.001	*p* < 0.001	*p* = 0.141	*p* = 0.389
1st	10.2 (9.3–11.1)	28.1 (25.9–30.3)	16.9 (15.6–18.2)	75.8 (71.1–80.5)	72.7 (68.4–77.1)	55.3 (51.6–59.0)
2–3rd	14.0 (12.8–15.2)	37.3 (34.3–40.4)	27.6 (25.5–29.7)	88.1 (82.9–93.3)	75.3 (70.7–80.1)	56.1 (52.4–59.8)
4th or more	13.4 (12.3–14.5)	37.7 (34.7–40.8)	28.8 (26.5–31.1)	91.9 (86.6–97.2)	78.1 (73.3–82.9)	57.7 (53.9–61.5)
Study shift	*p* = 0.237	*p* = 0.310	*p* = 0.383	*p* = 0.164	*p* = 0.104	*p* = 0.126
Day	12.6 (11.5–3.7)	34.4 (31.6–37.2)	24.8 (22.9–26.7)	84.4 (79.3–89.5)	72.9 (68.6–77.3)	53.9 (50.4–57.4)
Night	13.9 (12.7– 15.1)	37.3 (33.3–30.4)	27.1 (25.0–29.2)	90.1 (84.9–95.3)	79.1 (74.0–84.3)	60.7 (56.5–64.9)

**Table 4:** Multiple hierarchical logistic regression for demographic (block 1) and university setting (block 2) correlates of health risk behaviors of Brazilian university students ^a^.

	Personal security and violence					Sex behavior and contraception				
Correlates	Involvement in physical fight			Suicidal ideation		≥3 occasional partners per year			Don’t always use a protective barrier	
	OR_Adjusted (95%CI)_		*p*-value	OR_Adjusted (95%CI)_	*p*-value	OR_Adjusted (95%CI)_		*p*-value	OR_Adjusted (95%CI)_	*p*-value
Block 1—Demographic data										
Gender										
Female	Reference			1.69 (1.28–2.48)		Reference			Reference	
Male	2.82 (1.98–4.65)		0.001	Reference	0.023	1.66 (1.12–2.74)		0.016	1.54 (1.10–2.49)	0.012
Age										
≤20 years	2.29 (1.61–3.78)		0.001	Reference		Reference			1.50 (1.04–2.48)	0.032
21–25 years	1.58 (1.09–2.61)		0.024	1.21 (0.92–1.76)	0.096	1.55 (1.08–2.56)		0.027	1.28 (0.89–2.13)	0.107
≥26 years	Reference			1.48 (1.13–2.15)	0.036	1.59 (1.11–2.69)		0.013	Reference	
Block 2—University setting data										
Housing type										
Parent’s home	Reference					1.19 (0.83–1.97)		0.257	Reference	
Student’s residence	2.35 (1.63–3.89)		<0.001			1.51 (1.05–2.53)		0.030	1.52 (1.07–2.51)	0.027
Homestay	1.88 (1.31–3.11)		0.008			1.42 (0.99–2.35)		0.051	1.34 (0.93–2.24)	0.099
Single home	1.57 (1.04–2.74)		0.036			Reference			1.30 (0.89–2.17)	0.107
Academic year										
1st				Reference		Reference			1.48 (1.03–2.45)	0.046
2–3rd				1.14 (0.87–1.65)	0.114	1.47 (1.02–2.43)		0.048	1.32 (0.92–2.21)	0.098
4th or more				1.43 (1.09–2.08)	0.033	1.58 (1.10–2.61)		0.021	Reference	
	Addictive substances use						Eating habits, physical activity, and sleep			
Correlates	Smoking		Binge drinking		Illicit drugs		<5 servings per week of fruits and vegetables		Physical inactivity	
	OR_Adjusted (95%CI)_	*p*-value	OR_Adjusted (95%CI)_	*p*-value	OR_Adjusted (95%CI)_	*p*-value	OR_Adjusted (95%CI)_	*p*-value	OR_Adjusted (95%CI)_	*p*-value
Block 1—Demographic data										
Gender										
Female	Reference		Reference		Reference		Reference		1.67 (1.16–2.76)	0.021
Male	1.92 (1.34–3.21)	0.005	2.61 (1.81–4.35)	0.001	2.26 (1.57–3.78)	0.001	1.59 (1.11–2.63)	0.018	Reference	
Age										
≤20 years	Reference		Reference		Reference		Reference		Reference	
21–25 years	1.85 (1.28–3.07)	0.009	1.49 (1.04–2.46)	0.032	1.92 (1.33–3.17)	0.007	1.45 (1.02–2.40)	0.048	1.35 (0.92–2.23)	0.098
≥26 years	1.93 (1.34–1.24)	0.005	1.57 (1.09–2.59)	0.024	2.01 (1.40–3.32)	0.003	1.43 (1.00–2.36)	0.049	1.59 (1.11–2.64)	0.020
Block 2—University setting data										
Housing type										
Parent’s home	Reference		Reference		Reference		Reference			
Student’s residence	1.96 (1.36–3.24)	0.004	1.61 (1.12–2.67)	0.016	1.98 (1.36–3.28)	0.005	1.52 (1.06–2.51)	0.029		
Homestay	1.83 (1.27–3.02)	0.010	1.36 (0.95–2.25)	0.064	1.46 (1.01–2.41)	0.047	1.49 (1.04–2.46)	0.032		
Single home	1.32 (0.92–2.18)	0.099	1.27 (0.88–2.10)	0.109	1.42 (0.99–2.35)	0.051	1.34 (0.93–2.21)	0.099		
Campus size										
<1500 students			Reference		Reference		Reference			
1500–3000 students			1.28 (0.89–2.12)	0.107	1.26 (0.87–2.08)	0.112	1.29 (0.90–2.13)	0.110		
>3000 students			1.58 (1.10–2.61)	0.021	1.90 (1.32–3.14)	0.009	1.46 (1.01–2.41)	0.047		
Academic year										
1st			Reference		Reference		Reference		Reference	
2–3rd			1.56 (1.08–2.58)	0.029	1.94 (1.35–3.21)	0.004	1.48 (1.03–2.45)	0.045	1.43 (0.99–2.36)	0.051
4th or more			1.60 (1.11–2.64)	0.018	2.03 (1.41–3.35)	0.001	1.54 (1.07–2.54)	0.027	1.56 (1.08–2.58)	0.025
Study shift										
Day									Reference	
Night									1.47 (1.02–2.43)	0.046

^a^ *Odds ratio* adjusted by the other variables included in the model.

## Data Availability

The data used are available within the manuscript.

## References

[1] Zimmermann M., O’Donohue W., Vechiu C. (2020). A Primary Care Prevention System for Behavioral Health: The Behavioral Health Annual Wellness Checkup. J. Clin. Psychol. Med. Settings.

[2] (2018). World Health Statistics 2018: Monitoring Health for the SDGs, Sustainable Development Goals.

[3] Beaudry K.M., Ludwa I.A., Thomas A.M., Ward W.E., Falk B., Josse A.R. (2019). First-year university is associated with greater body weight, body composition and adverse dietary changes in males than females. PLoS ONE.

[4] Haas J., Baber M., Byrom N., Meade L., Nouri K. (2018). Changes in student physical health behaviour: An opportunity to turn the concept of a Healthy University into a reality. Perspect. Public Health.

[5] El Ansari W., Ssewanyana D., Stock C. (2018). Behavioral health risk profiles of undergraduate university students in England, Wales, and Northern Ireland: A cluster analysis. Front. Public Health.

[6] Murphy J.J., MacDonncha C., Murphy M.H., Murphy N., Timperio A., Leech R.M., Woods C.B. (2019). Identification of health-related behavioural clusters and their association with demographic characteristics in Irish university students. BMC Public Health.

[7] Peltzer K., Pengpid S., Mohan K. (2014). Prevalence of health behaviors and their associated factors among a sample of university students in India. Int. J. Adolesc. Med. Health.

[8] Shekari F., Habibi P., Nadrian H., Mohammadpoorasl A. (2020). Health-risk behaviors among Iranian university students, 2019: A web-based survey. Arch. Public Health.

[9] Ansari W.E., Khalil K.A., Ssewanyana D., Stock C. (2018). Behavioral risk factor clusters among university students at nine universities in Libya. AIMS Public Health.

[10] Amiri M., Raei M., Sadeghi E., Keikavoosi-Arani L., Khosravi A. (2023). Health-promoting lifestyle and its determining factors among students of public and private universities in Iran. J. Educ. Health Promot..

[11] Caballero L.G.R., Delgado E.M.G., López A.L.M. (2017). Prevalence of modifiable behavioral risk factors associated to non-communicable diseases in Latin American college students: A systematic review. Nutr. Hosp..

[12] Silva D.A.S., Petroski E.L. (2012). The simultaneous presence of health risk behaviors in freshman college students in Brazil. J. Community Health.

[13] American College Health Association-National College Health Assessment (ACHA-NCHA) (2020). Reference Group Data Report—Fall 2019.

[14] Kwan M.Y.W., Faulkner G.E.J., Arbour-Nicitopoulos K.P., Cairney J. (2013). Prevalence of health-risk behaviours among Canadian post-secondary students: Descriptive results from the National College Health Assessment. BMC Public Health.

[15] Zhang Y.B., Pan X.F., Chen J., Cao A., Xia L., Zhang Y., Wang J., Li H., Liu G., Pan A. (2021). Combined lifestyle factors, all-cause mortality and cardiovascular disease: A systematic review and meta-analysis of prospective cohort studies. J. Epidemiol. Community Health.

[16] Presidência da República (2010). Secretaria Nacional de Políticas sobre Drogas/SENAD. National Survey on the Use of Alcohol, Tobacco and Other Drugs among University Students in the 27 Brazilian Capitals.

[17] Oliveira C.S., Gordia A.P., Quadros T.M.B., Campos W. (2014). Physical activity of Brazilian university students: A literature review. Rev. Atenção Saúde.

[18] von Elm E., Altman D.G., Egger M., Pocock S.J., Gøtzsche P.C., Vandenbroucke J.P., STROBE Initiative (2007). The Strengthening the Reporting of Observational Studies in Epidemiology (STROBE) statement: Guidelines for reporting observational studies. Prev. Med..

[19] (2013). American College Health Association-National College Health Assessment II (ACHA): Reliability and Validity Analyses 2011.

[20] Rahn R.N., Pruitt B., Goodson P. (2016). Utilization and limitations of the American College Health Association’s National College Health Assessment instrument: A systematic review. J. Am. Coll. Health.

[21] Guedes D.P., Teixeira M. (2012). Semantic and conceptual equivalences of the National College Health Assessment II. Cad. Saude Publica.

[22] Guedes D.P., Silva A.L.S. (2020). National College Health Assessment II: Psychometric properties and concordance attributes of printed and online formats. Saude Pesq..

[23] Feroz U., Jami H., Masood S. (2015). Role of early exposure to domestic violence in display of aggression among university students. Pak. J. Psychol. Res..

[24] Straus M.A., Ramirez I.L. (2007). Gender symmetry in prevalence, severity, and chronicity of physical aggression against dating partners by university students in Mexico and USA. Aggress Behav..

[25] Liu C.H., Stevens C., Wong S.H.M., Yasui M., Chen J.A. (2019). The prevalence and predictors of mental health diagnoses and suicide among US college students: Implications for addressing disparities in service use. Depress. Anxiety.

[26] Jaesin S., Siyoung C.C., Beom-Young C.C., Jean-Philippe C., Jounghee L., Sungiae H. (2020). Sex and Racial/Ethnic Differences in Suicidal Consideration and Suicide Attempts among US College Students, 2011–2015. Am. J. Health Behav..

[27] Wang H., Long L., Cai H., Wu Y., Xu J., Shu C., Wang P., Li B., Wei Q., Shang X. (2015). Contraception and unintended pregnancy among unmarried female university students: A cross-sectional study from China. PLoS ONE.

[28] Ssewanyana D., Sebena R., Petkeviciene J., Lukács A., Miovsky M., Stock C. (2015). Condom use in the context of romantic relationships: A study among university students from 12 universities in four Central and Eastern European countries. Eur. J. Contracept. Reprod. Health Care.

[29] Bertoli R.S., Scheidmantel C.E., Carvalho N.S. (2016). College students and HIV infection: A study of sexual behavior and vulnerabilities. Braz. J. Sex. Transm. Dis..

[30] Larimer M.E., Kilmer J.R., Lee C.M. (2005). College student drug prevention: A review of individually-oriented prevention strategies. J. Drug Issues.

[31] Ministério da Saúde (2017). III National Survey on Drug Use by Brazilian Population (III LNUD).

[32] Jacob L., Freyn M., Kalder M., Dinas K., Kostev K. (2018). Impact of tobacco smoking on the risk of developing 25 different cancers in the UK: A retrospective study of 422,010 patients followed for up to 30 years. Oncotarget.

[33] Wicki M., Kuntsche E., Gmel G. (2010). Drinking at European universities? A review of students’ alcohol use. Addict. Behav..

[34] Hallett J., Howat P.M., Maycock B.R., McManus A., Kypri K., Dhaliwal S.S. (2012). Undergraduate student drinking and related harms at an Australian university: Web-based survey of a large random sample. BMC Public Health.

[35] Heradstveit O., Skogen J.C., Brunborg G.S., Lonning K.J., Sivertsen B. (2021). Alcohol-related problems among college and university students in Norway: Extent of the problem. Scand. J. Public Health.

[36] Richards D.K., Waddell J.T., Addictions Research Team (2023). Indirect associations between impulsivity and alcohol outcomes through motives for drinking responsibly among U.S. college students: An integration of Self-Determination Theory and the acquired preparedness model. Addict. Res. Theory.

[37] White H.R., McMorris B.J., Catalano R.F., Fleming C.B., Haggerty K.P., Abbott R.D. (2006). Increases in alcohol and marijuana use during the transition out of high school into emerging adulthood: The effects of leaving home, going to college, and high school protective factors. J. Stud. Alcohol..

[38] Peltzer K., Pengpid S. (2015). Nocturnal sleep problems among university students from 26 countries. Sleep Breath.

[39] Sa J., Choe S., Cho B.Y., Chaput J.P., Kim G., Park C.H. (2020). Relationship between sleep and obesity among U.S. and South Korean college students. BMC Public Health.

[40] Brinks H., Vincent G.E., Irwin C., Heidke P., Vandelanotte C., Williams S.L., Khalesi S. (2020). Associations between sleep and lifestyle behaviours among Australian nursing students: A cross-sectional study. Collegian.

[41] Turner R.W., Vissa K., Hall C., Poling K., Athey A., Alfonso-Miller P., Gehrels J.A., Grandner M.A. (2021). Sleep problems are associated with academic performance in a national sample of collegiate athletes. J. Am. Coll. Health.

[42] Irish L.A., Kline C.E., Gunn H.E., Buysse D.J., Hall M.H. (2015). The role of sleep hygiene in promoting public health: A review of empirical evidence. Sleep Med. Rev..

[43] Carone C.M.M., Silva B.P., Rodrigues L.T., Tavares O.S., Carpena M.S., Santos I.S. (2020). Factors associated with sleep disorders in university students. Cad. Saude Publica.

[44] Aune D., Giovannucci E., Boffetta P., Fadnes L.T., Keum N., Norat T., Greenwood D.C., Riboli E., Vatten L.J., Tonstad S. (2017). Fruit and vegetable intake and the risk of cardiovascular disease, total cancer and all-cause mortality—A systematic review and dose-response meta-analysis of prospective studies. Int. J. Epidemiol..

[45] Rodrigues V.M., Bray J., Fernandes A.C., Bernardo G.L., Hartwell H., Martinelli S.S., Lazzarin Uggioni P., Barletto Cavalli S., Proença R.P.D.C. (2019). Vegetable consumption and factors associated with increased intake among college students: A scoping review of the last 10 years. Nutrients.

[46] Ha E.J., Caine-Bish N. (2009). Effect of nutrition intervention using a general nutrition course for promoting fruit and vegetable consumption among college students. J. Nutr. Educ. Behav..

[47] Wardle J., Haase A.M., Steptoe A., Nillapun M., Jonwutiwes K., Bellisle F. (2004). Gender differences in food choice: The contribution of health beliefs and dieting. Ann. Behav. Med..

[48] Mirabitur E., Peterson K.E., Rathz C., Matlen S., Kasper N. (2016). Predictors of college-student food security and fruit and vegetable intake differ by housing type. J. Am. Coll. Health.

[49] Small M., Bailey-Davis L., Morgan N., Maggs J. (2013). Changes in eating and physical activity behaviors across seven semesters of college: Living on or off campus matters. Health Educ. Behav..

[50] Barnes S., Brown K., McDermott R., Bryant C.A., Kromrey J. (2012). Perceived parenting style and the eating practices of college freshmen. Am. J. Health Educ..

[51] Haase A., Steptoe A., Sallis J.F., Wardle J. (2004). Leisure-time physical activity in university students from 23 countries: Associations with health beliefs, risk awareness, and national economic development. Prev. Med..

[52] Keating X.D., Guan J., Piñero J.C., Bridges D.M. (2005). A meta-analysis of college students physical activity behaviors. J. Am. Coll. Health.

[53] (2023). Vigitel Brasil 2023: Surveillance of Risk and Protective Factors for Chronic Diseases by Telephone Survey: Estimates on the Frequency and Sociodemographic Distribution of Risk and Protective Factors for Chronic Diseases in the Capitals of the 26 Brazilian States and the Federal District in 2023.

[54] Bakker E.A., Lee D.C., Hopman M.T.E., Oymans E.J., Watson P.M., Thompson P.D., Thijssen D.H., Eijsvogels T.M. (2021). Dose-response association between moderate to vigorous physical activity and incident morbidity and mortality for individuals with a different cardiovascular health status: A cohort study among 142,493 adults from the Netherlands. PLoS Med..

[55] Meader N., King K., Moe-Byrne T., Wright K., Graham H., Petticrew M., Power C., White M., Sowden A.J. (2016). A systematic review on the clustering and co-occurrence of multiple risk behaviours. BMC Public Health.

[56] Eratay E., Aydoğan Y. (2015). Study of the relationship between leisure time activities and assertiveness levels of students of Abant Izzet Baysal University. Procedia Soc. Behav. Sci..

[57] Mielke G.I., Ramis T.R., Habeyche E.C., Oliz M.M., Tessmer S., Azevedo M.R., Hallal P.C. (2010). Physical activity and associated factors among first-year university students. Braz. J. Phys. Act. Health.

[58] Deforche B., Van Dyc D., Deliens T., De Bourdeaudhuij I. (2015). Changes in weight, physical activity, sedentary behaviour and dietary intake during the transition to higher education: A prospective study. Int. J. Behav. Nutr. Phys. Act..

[59] Bauman A.E., Reis R.S., Sallis J.F., Wells J.C., Loos R.J., Martin B.W. (2012). Correlates of physical activity: Why are some people physically active and others not?. Lancet.

